# A low-power VHF transceiver for airborne SAR with enhanced buried object detection using chirped signal processing

**DOI:** 10.1038/s41598-025-34246-2

**Published:** 2026-01-27

**Authors:** Yasser Siddik, Khalid F. A. Hussein, Hamada Esmaiel, Fathi E. Abd El-Samie, Ahmed S. Mubarak

**Affiliations:** 1 Electronics and Communications Department, Akhbar El Yom Academy , Cairo, Egypt; 2 Electrical Engineering Department, Faculty of Engineering, Aswan University, Aswan, Egypt; 3 Microwave Engineering Department, Electronics Research Institute (ERI), Cairo, 11843 Egypt; 4 Department of Electronics and Electrical Communications Engineering, Faculty of Electronic Engineering, Menoufia University, Menouf, 32952 Egypt

**Keywords:** Engineering, Mathematics and computing

## Abstract

A low-power airborne synthetic aperture radar (SAR) transceiver is presented for high-resolution detection of shallow buried structures, particularly underground tunnels. The system operates in the VHF band to exploit its strong ground-penetration capability, where the limited available bandwidth necessitates advanced waveform shaping to achieve sufficient imaging resolution. To address this challenge, an optimized piecewise-linear nonlinear frequency modulation (PWL-NLFM) chirp is designed using particle swarm optimization (PSO), jointly minimizing sidelobe levels while preserving the required pulse-compression ratio. The tunable parameter $Q$ controls the number of PWL segments, enabling a flexible trade-off between sidelobe suppression and pulse compression ratio according to mission requirements. Quantitative evaluation demonstrates that the proposed waveform significantly outperforms standard LFM and quadratic NLFM pulses, reducing the peak sidelobe level ratio (PSLR) to -33.0 dB and improving the integrated sidelobe ratio (ISLR) to -21.8 dB. A full two-dimensional (range–azimuth) point-target simulation further confirms that these improvements translate into superior SAR focusing, producing a cleaner and more isolated mainlobe with only slight broadening. This enhanced 2-D response increases the contrast between weak subsurface targets and background clutter, directly improving the detectability of tunnel features. The optimized PWL-NLFM waveform is integrated into a low-power SDR-based SAR transceiver, demonstrating its suitability for long-duration airborne sensing missions requiring deep penetration and high-contrast imaging.

## Introduction

**Fig. 1:**
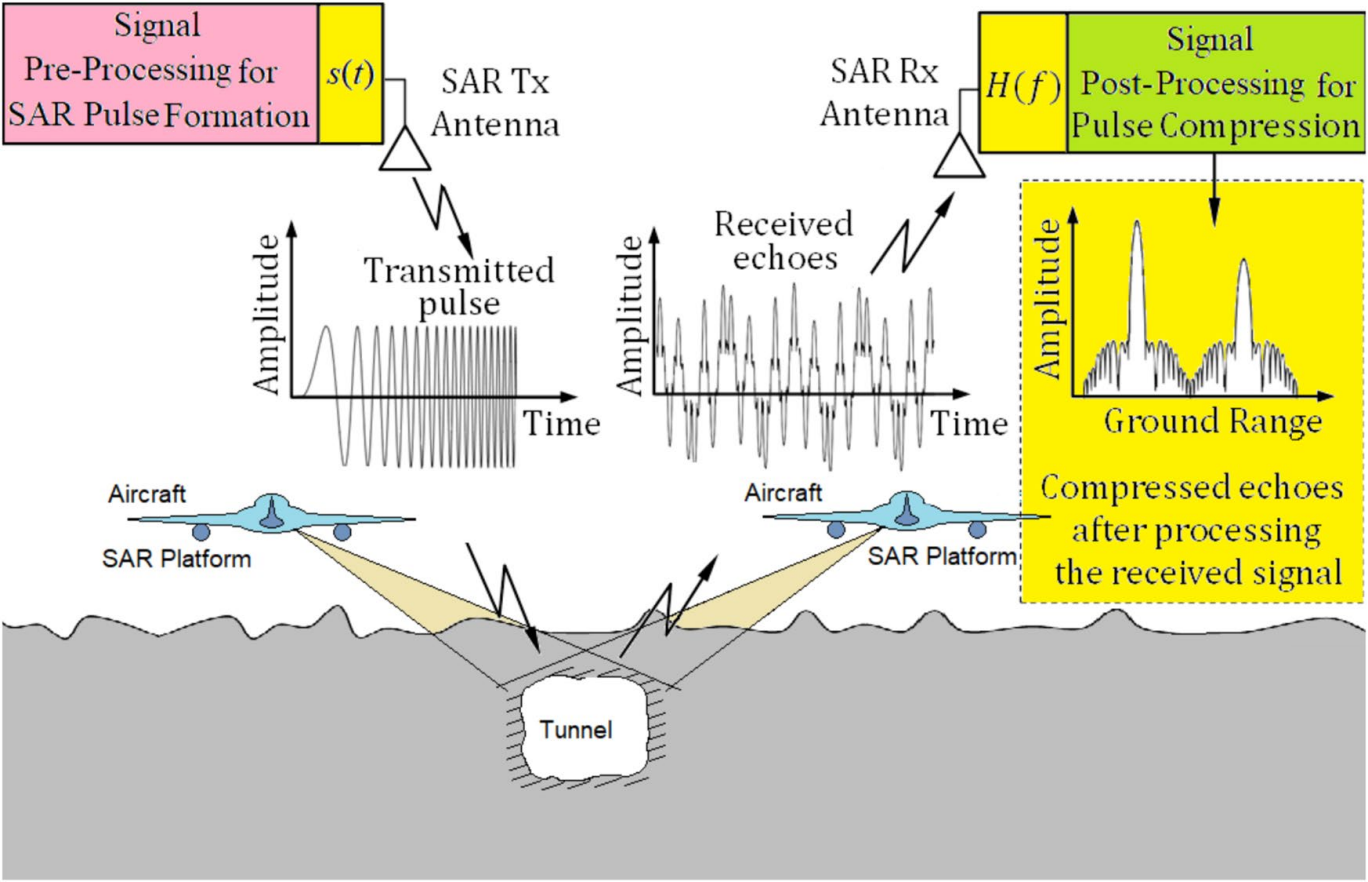
Scheme of pulse compression for low power operation and high imaging resolution in tunnel detection application using airborne SAR.

Synthetic Aperture Radar (SAR) is an important imaging technology for earth remote sensing that can operate independently of changing environmental conditions and imaging time. The crucial requirements for an efficient SAR system are achieving high resolution and improved target detection. To obtain high-resolution imaging, a shorter SAR pulse is needed, while maximizing the signal-to-noise ratio (SNR) requires a longer pulse duration to ensure greater pulse energy without increasing peak power ^1,2^. This introduces a trade-off between imaging resolution and detection performance. Hence, SAR pulse compression techniques are used to enhance imaging resolution without increasing the transmitted power ^3–6^. These techniques include shaping the SAR pulse with extended duration for better SNR, while simultaneously broadening the frequency bandwidth to emulate a shorter pulse, improving the resolution (Fig. 1). Linear frequency modulation (LFM) chirping is the most widely used pulse compression technique.

Recent research in SAR system design has focused mainly on developing SAR transceivers using software-defined radio (SDR) approaches ^7–13^. In SAR receivers applying pulse compression, the received echo is processed by a matched filter (MF). This filter outputs the echo as a pulse featuring a main lobe and lower-level sidelobes. One of the main functions of the MF in the SAR receiver is to maximize the signal-to-noise ratio (SNR), improving sensitivity and enhancing detection abilities. In addition, the MF suppresses the sidelobes in the received echo, contributing to higher imaging resolution. While processing an LFM chirped pulse, the MF response achieves a sidelobe level (SLL) of around −13 dB. Different methods have been suggested to reduce the SLL of LFM pulses, such as adaptive filtering ^14^, time and frequency windowing, and optimization techniques ^15^. Compared to conventional LFM, these approaches can lower the SLL, although they often lead to reduced SNR and a wider main lobe, resulting in a lower pulse compression ratio.

Another common approach in SAR systems is nonlinear frequency modulation (NLFM), aiming to achieve high-resolution imaging with acceptable SNR. Recent studies have introduced new SAR pulse compression schemes to improve detection performance and resolution. For example, a starring spotlight mode ^16^ was proposed to obtain high-resolution imaging with a low SLL. Here, NLFM was used for low PSLR in the range direction, and azimuth non-uniform sampling (ANUS) was used for low PSLR in the azimuth direction, leading to a compressed pulse with an SLL of −22 dB. Piecewise NLFM waveforms consisting of three subcarriers were suggested ^17^, yielding an SLL of − 27 dB. In another work ^18^, the authors used an orthogonal frequency-division multiplexing (OFDM) waveform, achieving an SLL of −28 dB. In another study ^19^, a piecewise NLFM method was introduced based on dividing the pulse into three segments, where the first and third segments used LFM and the second used NLFM, achieving an SLL of − 36.6 dB. An optimized approach using the Lagrangian method ^20^ resulted in an SLL of −38 dB. The advanced NLFM techniques ^21,^ ^22^, optimized with genetic algorithms, achieved SLL values of −40 dB and −40.6 dB, respectively.

In addition to these recent developments, a related contribution was presented ^23^. This work developed an intermediate-frequency NLFM signal generator specifically designed for UAV-borne SAR missions. The introduced method focused on hardware implementation of the NLFM at the intermediate frequency (IF) stage to improve the real-time signal generation capability. Compared with that study, our work differs in three key aspects: (i) we target a VHF airborne SAR system aimed at buried-object and tunnel detection, where low-power operation and ground-penetration capability dominate system requirements; (ii) instead of relying on a fixed NLFM generation hardware, we employed an optimized piecewise-linear (PWL) time–frequency trajectory whose slopes are determined using particle swarm optimization to jointly minimize the SLL and achieve the desired pulse-compression ratio (PCR); and (iii) our SAR transceiver integrates the optimized waveform into a low-power software-defined architecture tailored for long-duration airborne sensing. This comparison clarifies the complementary nature of the two approaches and highlights the novelty of the proposed method in the context of VHF SAR waveform optimization.

Electromagnetic operation in the VHF band is particularly significant for the intended application of shallow buried-object and underground-tunnel detection. RF waves in this frequency range exhibit reduced attenuation in soil and subsurface media, allowing deeper ground penetration than microwaves. At the same time, VHF systems operate under strict bandwidth constraints, which limit the achievable range resolution and place additional emphasis on efficient waveform shaping. This motivates the development of our optimized piecewise NLFM waveform, designed to maximize resolution and suppress sidelobes while respecting the bandwidth and power limitations inherent in VHF SAR platforms. Thus, the proposed method directly addresses the challenges of low-frequency airborne ground-penetrating radar.

In contrast to conventional NLFM techniques that produce a fixed trade-off between SLL and mainlobe width, the proposed PWL-NLFM method introduces a tunable design parameter, the number of frequency segments $Q$. By adjusting $Q$, the designer can continuously control the balance between sidelobe suppression and PCR, allowing the waveform to be tailored to the operational needs of VHF ground-penetrating SAR missions. A larger $Q$ yields stronger sidelobe suppression for high-contrast detection of buried objects, whereas smaller $Q$ preserves a narrower impulse response for improved range resolution. This tunability represents a key distinguishing feature of the proposed method compared with existing NLFM schemes that offer only fixed performance points.

This paper presents a novel method to reduce the PSLR of radar pulses, aiming at improving the imaging resolution and detection performance in SAR systems. The proposed method uses an arbitrarily defined piecewise linear (PWL) curve to shape the instantaneous frequency over the pulse duration. Particle swarm optimization (PSO) is used to optimize the slopes of these linear segments ^24^ to achieve two objectives: minimizing the SLL and yielding the desired pulse compression ratio (PCR). A computationally efficient PSO algorithm is designed to ensure rapid convergence with only a few iterations required to reach a stable solution. In this algorithm, the control parameters are the slopes of the linear segments, which determine the position of each particle in the swarm. The proposed technique functions effectively as an optimized NLFM for SAR pulse compression. The developed algorithm is highly efficient and converges quickly. The new SAR pulse compression technique is utilized in the implementation of a software-defined transceiver for the SAR system.

The conceptual SDR-based design of the proposed SAR system transceiver is explained in the next section of the paper. Sect. “Frequency Chirping of the SAR Pulse” gives an explanation of the frequency modulation for chirping the SAR pulse, and Sect. “Preprocessing Algorithm for SAR Pulse Formation and Construction of MF Transfer Function” gives an explanation of the preprocessing algorithm for SAR pulse formation and construction of the MF transfer function. Sect. “Optimized Time-Frequency Curve for High Resolution and Enhanced Performance” describes the application of PSO to construct the optimized shape of the time–frequency curve for frequency modulation of the SAR pulse. Sect. “Results and Discussion” gives a discussion of the numerical results and highlights the conclusions of the paper.

## Conceptual SDR-based design of the proposed SAR system transceiver

The proposed algorithms needed for SAR pulse formation in the transmitter and for construction of MF in the receiver are described in this section. In addition, the SDR transceiver design based on this method is discussed.

### Transmitter design

**Fig. 2:**
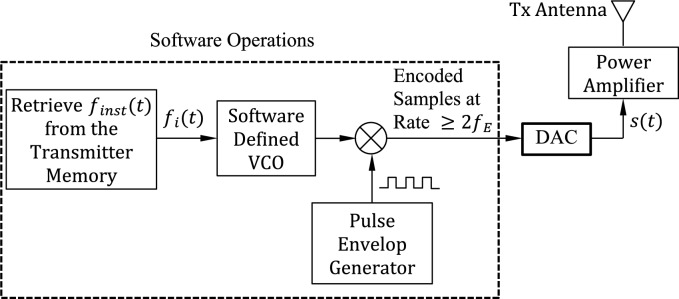
The proposed software-defined transmitter of the high-resolution SAR system.

A software-defined SAR transmitter has all its functionalities applicable as software modules except for the fast digital-to-analog converter (DAC) and the following power amplifier (PA) as shown in Fig. 2. In the transmitter, the optimized time–frequency curve, ${f}_{inst}(t)$, that has been constructed in the preprocessing stage described in Sect. “LFM Chirping of the SAR Pulse” is retrieved from the transmitter memory. The instantaneous frequency is transformed to a voltage signal to be the input of a software-defined VCO, which, in turn, generates a sinusoidal signal whose frequency is proportional to the input voltage. The transmitted SAR pulse, $s\left(t\right)$, is constructed in the transmitter by multiplying the output of the VCO by the output of a pulse envelop generator that is responsible for determining the pulse repetition rate, pulse duration, and pulse amplitude (pulse energy).

### Receiver design

**Fig. 3:**
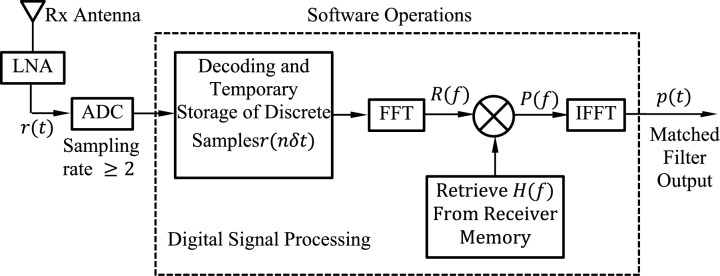
The proposed software-defined MF receiver of the high-resolution SAR system.

**Fig. 4:**
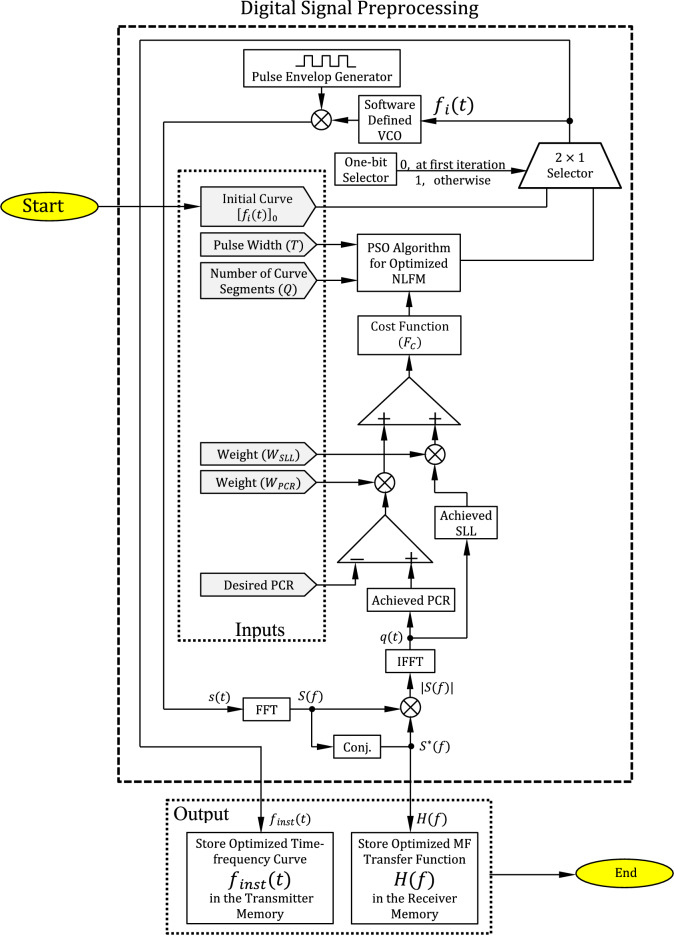
Block diagram of the proposed digital signal processing algorithm for construction of the transmitted SAR pulse and the matched filter transfer function for the design of the proposed low-power transceiver.

The transfer function of the MF is given by $H(f)$ that has been constructed and stored in the receiver memory using the preprocessing algorithm explained in Sect. “Inputs to the Optimization Algorithm” and illustrated in the block diagram shown in Fig. 2. The proposed receiver is presented in Fig. 3. A software-defined SAR receiver has all its functionalities applicable as software modules except for the low-noise amplifier (LNA) and the fast analog-to-digital converter (ADC) shown in Fig. 4. The received signal is fed into a MF whose transfer function is the conjugate of the transmitted signal as given by (8). The signal, $p\left(t\right)$, at the output of the MF is the compressed pulse, which is the inverse Fourier transform (IFFT) of the product of the received signal spectrum, $R(f)$, and the transfer function of the MF, $H\left(f\right)$. Thus, the signal spectrum at the MF output can be expressed as follows.

1 $$P\left(f\right)=H\left(f\right) R(f)$$

It can be assumed that the received pulse $r(t)$ is identical to that of the transmitted signal, $s\left(t\right)$, except for the separable modifications of the magnitude and phase due to scattering on the SAR target. Thus, one can write the following expression for the spectrum of $r(t)$.

2 $$R\left(f\right)= {A}_{0}{e}^{j{\varphi }_{0}}S\left(f\right)$$

where ${A}_{0}$ and ${\varphi }_{0}$ are the magnitude and phase of the coefficient of backscattering on the SAR target. Thus, the frequency-domain expression at the MF output can be expressed as,

3 $$P\left(f\right)={\left|{S\left(f\right)}^{ }\right|}^{2} {A}_{0}{e}^{j{\varphi }_{0}}$$

From (1), it is shown that the bandwidth of the processed echo pulse, $p\left(t\right)$, at the MF output is the same as that of the transmitte chirped pulse, $s\left(t\right)$.

## Frequency chirping of the SAR pulse

This paper presents a novel design of SAR transceiver to achieve low power consumption, high imaging resolution and enhanced detection performance.

### LFM chirping of the SAR pulse

Frequency chirping using LFM is the conventional method for radar pulse compression, where the instantaneous frequency increases linearly with the time over the SAR pulse duration to improve the resolution of the radar. The radar pulse is constructed as a sinusoidal signal whose amplitude is constant over the pulse duration and zero otherwise. The frequency of the sinusoidal signal is ${f}_{B}$ at the beginning of radar pulse and increases linearly with time until it reaches ${f}_{E}$ at the end of the pulse. If the pulse duration is $T,$ then the slope of increase of the instantaneous frequency is $\left({f}_{E}-{f}_{B}\right)/T$. The LFM chirping of the SAR pulse achieves PCR of $127$ and SLL of $-13\text{ dB}$.

### Second-order NLFM chirping of the SAR pulse

In second-order NLFM chirping of the SAR pulse, the instantaneous frequency has a quadratic dependence on the time. During the pulse transmission, the RF transmitted signal is expressed as follows:

4 $$s\left(t\right)=\mathrm{sin}{\theta }_{i}\left(t\right)$$

where ${\theta }_{i}\left(t\right)$ is the instantaneous value of the angle.

The instantaneous frequency, ${f}_{i}(t)$ of the RF transmitted signal, can be acquired by differentiating ${\theta }_{i}\left(t\right)$ with respect to the time,

5 $${f}_{i}\left(t\right)=\frac{1}{2\pi }\frac{\partial {\theta }_{i}\left(t\right)}{\partial t}$$

To acquire second-order NLFM chirping, the instantaneous frequency, ${f}_{i}(t)$, should take the following form,

6 $${f}_{i}\left(t\right)=A({t-{T}_{B})}^{2}+\frac{B-A{T}^{2}}{T} \left(t-{T}_{B}\right)+\left({f}_{C}-\frac{B}{2}\right), {T}_{B}\le t\le {T}_{E}$$

where $A$ is the quadratic coefficient controlling the nonlinearity, ${f}_{C}$ is the center frequency of sweep, which is the operational frequency of the SAR system, $B$ is the bandwidth, which is the difference between the beginning and the end frequencies, ${f}_{B}$ and ${f}_{E}$, respectively, thus $B={f}_{E}-{f}_{B}$, ${T}_{B}$ is the beginning time of the pulse, ${T}_{E}=T+{T}_{B}$ is the end time of the pulse, and $T$ is the pulse duration.

The start frequency of sweep ${f}_{B}$ is obtained by substituting $t={T}_{B}$ in (6),

7 $${f}_{B}={f}_{i}\left({T}_{B}\right)={f}_{C}-\frac{B}{2}$$

The end frequency of sweep ${f}_{E}$ is obtained by substituting $t={T}_{E}={T}_{B}+T$ in (6),

8 $${f}_{E}={f}_{i}\left({T}_{E}\right)={f}_{C}+\frac{B}{2}$$

The formulation of (6) can be supported by the references ^25–27^, commonly used for NLFM waveform synthesis.

The angle ${\theta }_{i}\left(t\right)$ can be expressed as ${\theta }_{i}\left(t\right)=\int {f}_{i}\left(t\right) dt$; this gives,

9 $${\theta }_{i}\left(t\right)=2\pi \left[\frac{A}{3}{(t-{T}_{B})}^{3}+ \frac{B-A{T}^{2}}{2T}{(t-{T}_{B})}^{2}+{f}_{B}(t-{T}_{B})+c\right], {T}_{B}\le t\le {T}_{E}$$

where $c$ is the constant of integration and is equal to $\theta \left({t}_{B}\right)$ i.e. the angle at the beginning time of the SAR pulse.

To obtain the phase of the sinusoidal signal equal to zero at the beginning time of each pulse, one should set $c=0$. In this case, $\theta \left(t\right)$ can be expressed as follows.

10 $${\theta }_{i}\left(t\right)=\frac{2\pi A}{3}{(t-{T}_{B})}^{3}+ \frac{\pi (B-A{T}^{2})}{T}{(t-{T}_{B})}^{2}+2\pi {f}_{B }(t-{T}_{B}), {T}_{B}\le t\le {T}_{E}$$

### Time discretization for simulation

For simulation of the SAR pulse transmission, reception, and processing, the time should be discretized to apply fast Fourier transform (FFT) and inverse fast Fourier transform (IFFT) operations. If $N$ is the number of time samples of the transmitted SAR pulse, $s\left(t\right)$ and the time interval between the successive samples is $\updelta t$ then, the start time of the ${n}^{\mathrm{th}}$ sampling period is ${t}_{n}=(n - 1)\delta t + {T}_{B}$ and ${T}_{B}=({n}_{B}-1)\delta t$, where ${n}_{B}$ is the index of the time sample at which the transmitted pulse starts. Thus, the total time for simulation is ${T}_{T }=L\delta t$, where $L$ is the total number of sampling periods over which the simulation is performed. The center frequency, ${f}_{C}=\left({f}_{B}+{f}_{E}\right)/2$, is the operating frequency of the SAR. The bandwidth of operation is B = ${f}_{E}-{f}_{B}$, then the start and stop frequencies ${f}_{B}$ and ${f}_{E}$ can be, respectively, expressed as follows.

11 $${f}_{B}={f}_{C}-\frac{B}{2}, {f}_{E}={f}_{C}+\frac{B}{2}$$

The ${n}^{\mathrm{th}}$ frequency component of the transmitted pulse spectrum (i.e. its Fourier transform) can be expressed as,

12 $${f}_{n }={f}_{B}+\left(n - 1\right)\updelta f, \delta f=\frac{1}{L\updelta t}$$

For accurate simulation, the sampling frequency, ${f}_{S}$, should be much greater than twice the end frequency ${f}_{E}$, i.e. ${f}_{S}\ge 2{f}_{E}$.

## Preprocessing algorithm for SAR pulse formation and construction of MF transfer function

In this paper, an optimized arbitrarily-shaped staircase time–frequency curve is proposed for NLFM for radar pulse compression as a step for the complete design of a low-power and enhanced-performance SAR transceiver. Due to the proposed transceiver design, the SAR system can operate in the VHF range for detection of shallow hidden tunnels with enhanced detection performance and high resolution. In this section, the proposed pre-processing algorithms for construction of the optimized SAR pulse compression and the matched filter transfer function are discussed. The block diagram of the proposed digital signal pre-processing that is a prerequisite for the construction of the proposed SAR transceiver is presented in Fig. 4.

### Inputs to the optimization algorithm

The pulse design parameters are the inputs to the PSO algorithm including the SAR pulse width, the number of linear segments, $T$, $Q$, of the staircase time–frequency curve, and the weights required to formulate the cost function which is constructed as the summation of two terms. The first is the calculated SLL multiplied by a weight factor ${W}_{SLL}$, and the second is the difference between the achieved and the desired PCR, which is multiplied by the weight factor ${W}_{PCR}$.

### Formation of the SAR Pulse for the transmitter

In the transmitter, the transmitted SAR pulse, $s\left(t\right)$, is made by help of the PSO algorithm where the instantaneous frequency calculated by the PSO algorithm is transferred to a voltage signal to be the input of a voltage-controlled oscillator (VCO). This VCO generates a sinusoidal signal, the frequency of which is proportional to the input voltage. The output of this VCO is then multiplied by the output of a pulse envelop generator which is responsible for determining the pulse repetition rate, pulse amplitude, and pulse duration. The time–frequency curve, ${f}_{i}(t)$, is saved in the transmitter memory with the proper sampling rate.

### Construction of the matched filter transfer function for the receiver

To get its spectrum in the frequency-domain, $S(f)$, the FFT operation is applied to the chirped SAR pulse, $s(t)$. The transfer function, $H(f)$, of the matched filter is then calculated as the conjugate of $S(f)$.

13 $$H\left(f\right)={S}^{*}(f)$$

$\delta f$ separates the frequency samples of $H(f)$ and it is determined by the FFT as $\delta f={\left(L \delta t\right)}^{-1}$. The frequency samples of $H(f)$ (both magnitude and phase) are saved in the memory of the SAR receiver.

## Optimized time–frequency curve for high resolution and enhanced performance

The optimization method of the time–frequency curve used to minimize the SLL of the and to get a desired value of the PCR is described in details in this section. First, the instantaneous frequency dependence on the time is formulated as a curve that is arbitrarily shaped close to that of a quadratic equation with PWL segments. Then, the PSO algorithm is applied to fulfill the optimization goals by arriving at the optimum time–frequency curve.

### Proposed frequency modulation scheme for SAR pulse chirping

The staircase segmentation of the instantaneous frequency as a function of time results in a type of non-linear frequency modulation for SAR pulse compression with varying sweep rates. The frequencies of the successive time intervals of LFM linearly sweep with the given time frame.

If $Q$ is the number of segments constructing the staircase curve and $\Delta t$ is the duration of each segment then, the start and end times of the ${q}^{th}$ linear segment are ${t}_{q-1}$ and ${t}_{q}$.

The time duration of the SAR transmitted pulse is expressed as follows,

14 $$T={t}_{Q}-{t}_{0}=Q\Delta t$$

where,

15 $${t}_{0}={T}_{B}$$

The frequency ${f}_{1}$ at the end of the first time interval $\Delta t$ is expressed as follows

16 $${f}_{1}={s}_{1}\Delta t+{f}_{0}$$

Then, the slope ${s}_{1}$ of the first linear segment is expressed as follows

17 $${s}_{1}=\frac{{f}_{1}-{f}_{0}}{\Delta t}$$

The slope ${s}_{q}$ of the ${q}^{th}$ segment of the time–frequency curve is expressed as,

18 $${s}_{q}=\frac{{f}_{q}-{f}_{q-1}}{{t}_{q}-{t}_{q-1}}=\frac{{f}_{q}-{f}_{q-1}}{\Delta t}$$

Then, the ${q}^{th}$ frequency component of the chirped SAR pulse is expressed as,

19 $${f}_{q}={f}_{q-1}+{s}_{q}\Delta t$$

**Fig. 5:**
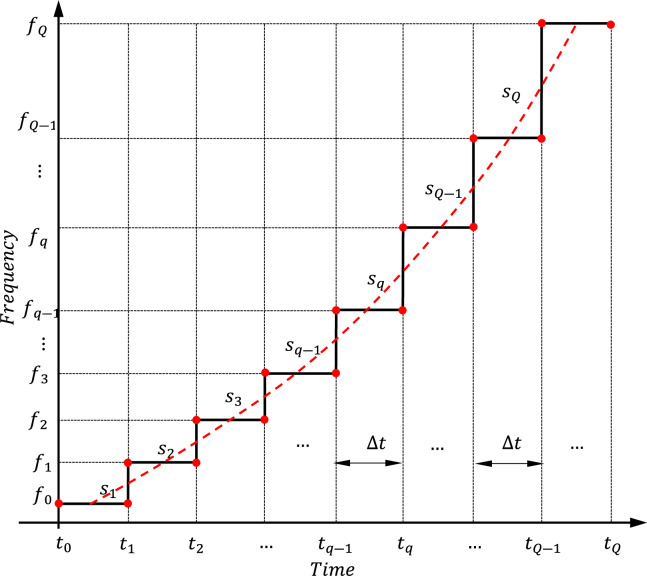
Staircase (piecewise horizontally segmented) curve for the dependence of the instantaneous frequency on the time.

Figure 5 presents the PWL curve which describes the instantaneous frequency ${f}_{inst}$ as a function of the time. The relation should be continuous, which means that the successive step segments of the curve should be connected, while the slopes {${s}_{1}$, ${s}_{2}$,.., ${s}_{Q}$} can be varied to minimize the SLL for a desired PCR. The PSO method is here applied to adjust the shape of the curve describing the instantaneous frequency dependence on the time. This is accomplished by selecting the best values of the slopes {${s}_{1}$, ${s}_{2}$,.., ${s}_{Q}$} to achieve the optimization goals. It should be noticed that $Q$ has a great effect on the results of optimization.

### Application of PSO for minimization of the SLL of the processed echo pulse

The purpose of the optimization problem is to arrive at the optimal shape of the time–frequency curve. It should be considered that the linear segments slope continuously linked to form the curve are the parameters that determine each particle position in the swarm. It is needed to arrive at the proper values of the slopes of the linked linear segments to achieve the optimization goals which are satisfying the required PCR and minimizing the SLL of the received pulse at the MF output.

#### Formulation of the optimization problem

In the optimization problem, each particle of the swarm has a position that is determined by a set of the slopes {${s}_{1}$, ${s}_{2}$,.., ${s}_{Q}$}$.$ The swarm has 15 particles. The initial position of each particle is set to realize the instantaneous frequency dependence on the time linearly with little random perturbation to obtain the initial curves representing the particle positions different from each other.

The aim of the PSO algorithm is to minimize the cost function which is formulated as follows.

20 $${\mathcal{F}}_{\cos t} = W_{SLL} l + W_{T} \, |T_{A} - T_{D} |$$

where, $l$, is the obtained SLL, ${T}_{A}$ is the obtained pulse width while ${T}_{D}$ is the desired pulse width, ${W}_{SLL}$ is the weight factor of the SLL and ${W}_{T}$ is the weight factor of the deviation of the obtained pulse width from the desired pulse width. The cost function can now be alternatively formulated as shown in the block diagram of the digital signal preprocessing in Fig. 4 to obtain the following expression.

21 $${\mathcal{F}}_{\cos t} = W_{SLL} l + W_{PCR} \, |C_{A} - C_{D} |$$

where ${C}_{A}$ is the obtained PCR, while ${C}_{D}$ is the desired PCR, and ${W}_{PCR}$ is the weight factor of the deviation of the obtained PCR from the desired one.

It may be worthwhile to mention that Eq. (20) uses the pulse width deviation $\left|{T}_{A}-{T}_{D}\right|$ weighted by ${W}_{T}$, while Eq. (21) uses the pulse compression ratio deviation $\left|{C}_{A}-{C}_{D}\right|$ weighted by ${W}_{PCR}$. Since the pulse width and the PCR are inversely related and have different orders of magnitude, the corresponding weights ${W}_{T}$ and ${W}_{PCR}$ cannot be numerically identical, even though they both serve to balance the second term in the cost function.

The PSO algorithm runs iteratively in order to get the best values for the slopes of the PWL segments forming the time–frequency curve. The particle position in each iteration is determined by the N-dimensional vector given by the set of slopes {${s}_{1}$, ${s}_{2}$,.., ${s}_{Q}$}. The process shown in the block diagram in Fig. 4 is employed to get the shape of the received SAR pulse at the output of the receiver $p\left(\it{t}\right)$. However, to form the chirped transmitted pulse, the optimization process requires feedback from the received pulse, $p\left(\it{t}\right)$. As this pulse has not been achieved, a simulated version of $p\left(\it{t}\right)$ is obtained in the transmission. This is done by including a MF in the transmitter, that is identical to that of the receiver. The simulated version of $p\left(\it{t}\right)$ is $q\left(\it{t}\right)$, from which, the SLL and pulse can be calculated to give the inputs needed for the PSO algorithm as shown in Fig. 4.

The PSO algorithm runs iteratively to determine the optimal slopes of the PWL segments forming the time–frequency curve. The particle position in each iteration is represented by the N-dimensional vector {${s}_{1}$, ${s}_{2}$,.., ${s}_{Q}$}. The process illustrated in the block diagram of Fig. 4 is used to model the shape of the received SAR pulse $p\left(t\right)$ at the output of the receiver. Since the actual received pulse is not available during optimization, a simulated version of $p\left(t\right)$, denoted $q\left(t\right)$, is generated within the transmitter preprocessing stage. This simulation applies a matched filter identical to the candidate transmitted waveform. The resulting $q\left(\it{t}\right)$ is used solely for evaluation of the sidelobe level (SLL) and pulse compression ratio (PCR) for each candidate particle, providing the necessary feedback to the PSO cost function. It is important to note that $q\left(t\right)$ is part of the optimization loop and is not physically transmitted.

#### Implementation of the PSO algorithm

The swarm of particles in the PSO moves progressively towards the optimization goal. During this movement, each particle position changes by adjusting the particle velocity that varies according to its past experience and the feedback received from its neighboring particles. Hence, each solution in the PSO algorithm is considered as a particle and each particle has a cost value. These cost values can be calculated using the cost function formulated to reduce the cost and approach the optimization goals. All those particles conserve their individual best performance, in addition they know the best performance of their group. Each particle optimizes its velocity, taking in consideration its best performance and the best performance of the best particle in the swarm. There are two tactics of the PSO algorithm called global tactics and local tactics. Each particle in the global tactics tracks the best particle position of the swarm. While in the local tactics, it tracks its own optimal position.

PSO algorithm implementation can be divided into four stages: the first is initialization of positions,${{\mathbf{x}}_{\nu }}^{(0)}$ and velocities,${{\mathbf{v}}_{\nu }}^{(0)}$, of the particles of the swarm. Second is calculation of the local best position,${{\mathbf{y}}_{\nu }}^{(\tau )}$ for each particle in the present iteration, while third is calculation of the global best position, g, among the positions of all the particles in the present iteration. Fourth is calculation of the velocities, ${{\mathbf{v}}_{\nu }}^{(\tau +1)}$, and positions, ${{\mathbf{x}}_{\nu }}^{(\tau +1)}$, of the particles for the next iteration.

The particle velocity and position of the ${\nu }^{\mathrm{th}}$ in the ${\tau }^{\mathrm{th}}$ iteration of an iterative PSO algorithm are calculated using these equations:

22 $${{{\boldsymbol{v}}}_{\nu }}^{(\tau )}= u {{{\boldsymbol{v}}}_{\nu }}^{(\tau -1)}+ {c}_{1}{r}_{1} \left[{{{\boldsymbol{y}}}_{\nu }}^{\left(\tau -1\right)}- {{{\boldsymbol{x}}}_{\nu }}^{\left(\tau -1\right)}\right]+ {c}_{2}{r}_{2} [{{\boldsymbol{g}}}^{\left(\tau -1\right)} - {{{\boldsymbol{x}}}_{\nu }}^{\left(\tau -1\right)}]$$

23 $${{{\boldsymbol{x}}}_{\nu }}^{(\tau )}={{{\boldsymbol{x}}}_{\nu }}^{(\tau -1)}+{{{\boldsymbol{v}}}_{\nu }}^{(\tau )}$$

where $\tau$ is the iteration number (time index) while $u$ is the inertia weight parameter,${c}_{1},$ ${c}_{2}$ are acceleration factors and ${r}_{1},{r}_{2}$ are random numbers between 0 and 1.

##### Initialization of the particles positions

**Fig. 6:**
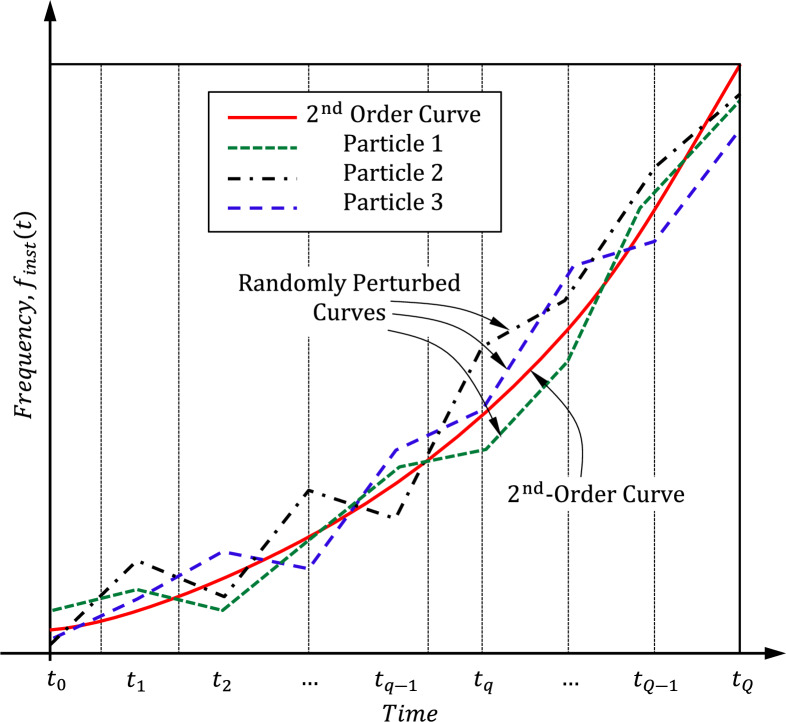
Initialization of the time–frequency curve corresponding to the different particles of the swarm as 2nd-order curve with random perturbations.

Each particle position can be initialized by conveying a set of initial values to the slopes ${{s}_{1}}^{\left(\nu ,0\right)}$, ${{s}_{2}}^{\left(\nu ,0\right)}$,.., ${{s}_{q}}^{\left(\nu ,0\right)}$,.., ${{s}_{Q}}^{\left(\nu ,0\right)}$, these initial values are determined by initializing the time–frequency curve corresponding to each particle as a second-order frequency dependence on time with small random perturbations as shown in Fig. 6. The 2nd-order time frequency curve is described by Eq. (6). Here the parameter $A$ is set to the value that produces the best performance of a ${2}^{\mathrm{nd}}$-order NLFM pulse chirping as regards the SLL from a previous knowledge or by applying a simple optimization process. Then small random perturbations are added to each slope ${{s}_{q}}^{\left(\nu ,0\right)}$ to get the initial positions of the swarm particles different from each other.

24 $${{S}_{q}}^{\left(\nu ,0\right)}= {S}_{q}+ {r}_{\nu }$$

Without these random perturbations, all the particles of the swarm will have the same position, and this causes the iterations of the PSO to diverge. Yet, during the application of the random perturbations, the linear segments of the piecewise linear time frequency curve should be continuous, which means that the start point of the ${q}^{\mathrm{th}}$ linear segment should coincide with the end point of the ${(q-1)}^{\mathrm{th}}$ linear segment. The local best position of each particle,${{\mathbf{y}}_{\nu }}^{(0)}$, is initially assigned the same initial value of the particle position, ${{\mathbf{x}}_{\nu }}^{(0)}$. The initial value of each particle velocity is set to zero; ${{{\boldsymbol{v}}}_{\nu }}^{(0)}=0$.

##### Progressive iterations of the PSO algorithm

The local best position, for each particle ${\mathbf{y}}_{\nu }^{(\tau )},$ is the position of this particle that gives the minimum value of the cost function throughout the progressive iterations in the PSO algorithm. For the particles in the swarm, the global best position, ${\mathbf{g}}^{(\tau )}$, is the position among the local best positions that gives the absolute minimum value of the cost function throughout the successive iterations in the PSO algorithm. For each particle in the swarm, velocity, ${\mathbf{v}}_{\nu }$, and position, ${\mathbf{x}}_{\nu }$, are updated in each iteration, as given by (19) and (20). Figure 6. shows the initialization of the particles positions before running the iterations of the PSO algorithms.

### Evaluation of the maximum SLL and PCR

**Fig. 7:**
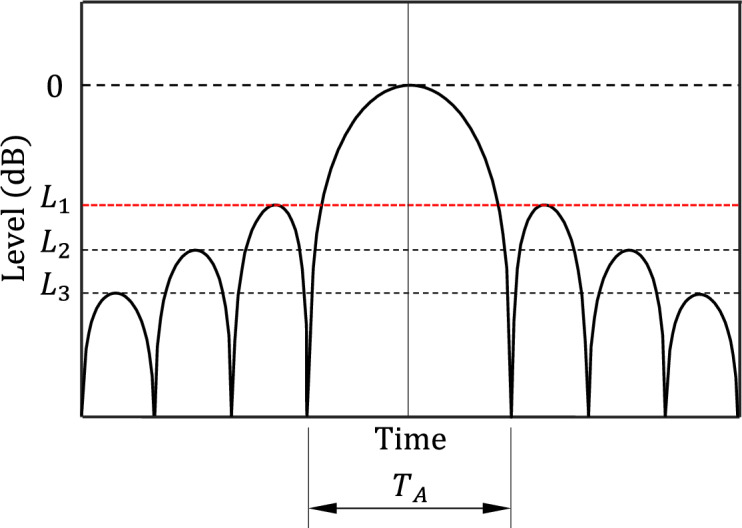
The normalized pulse, $p\left(t\right)$ at the output of the MF of the SAR system receiver.

At the output of the SAR receiver, the processed echo signal takes the time domain form sketched in Fig. 7. The pulse is normalized and plotted using decibel scale and the compressed pulse width can be calculated as the width between the two nulls on both sides of the main lobe as shown in Fig. 7. The maximum level of lobes excluding the main lobe is equal to the SLL. Usually the second lobe (the first side lobe) has the maximum level among all side lobes. Thus, the maximum SLL is ${L}_{1} (\mathrm{dB})$ and the compressed pulse width is ${T}_{A}$ while the PCR is defined as follows.

25 $$\eta =\frac{T}{{T}_{A}}$$

The aim of the proposed SAR receiver design as a SDR is to minimize the maximum SLL and to achieve the desired PCR.

### Power reduction factor

The aim of pulse compression is to transmit a long duration high energy pulse, but to detect a short duration pulse in the receiver filter output response to one or at most two radar range bins.

As the frequency amplitude increasing sinusoidal is maintained constant throughout the duration of the chirped rectangular pulse the maximum instantaneous power can be evaluated as follows.

26 $$P=\frac{1}{2}{A}^{2}$$

where the amplitude of the sinusoidal signal over the pulse duration is $A$, thus, the energy of the transmitted pulse can be expressed as follows

27 $${E}_{Tx}=P T=\frac{1}{2}{A}^{2}T$$

To get the same resolution of the SAR without obtaining the pre-processing and the post-processing as shown above, the time duration of the transmitted rectangular pulse would be ${T}_{A}$ while to achieve the same detection performance, the energy of this narrow pulse would be the same as that of chirped pulse. This can be expressed as follows.

28 $${{P}_{A} T}_{A}={E}_{Tx}$$

Using (25) and (27) the power reduction factor due to the processes done in the SAR transceiver can be formulated as follows.

29 $$\frac{P}{{P}_{A}}=2\frac{{T}_{A}}{T}=\frac{2}{\eta }$$

This means that due to the proposed transceiver design, the power is reduced by a factor that is double the inverse of the PCR, $\eta$.

### Point-target simulation and imaging metrics evaluation

To rigorously verify the quality of the optimized transmitted signal, we extend the analysis beyond one-dimensional waveform inspection and incorporate a full two-dimensional (range–azimuth) point-target simulation representative of actual SAR imaging conditions. This allows evaluation of the focusing performance obtained when using the proposed piecewise NLFM waveform, and provides quantitative comparisons of PSLR, ISLR, and IRW under different chirp strategies. Unlike the one-dimensional pulse-compression results of Sect. “Evaluation of the Maximum SLL and PCR”, this section verifies the complete SAR processing chain, including range compression, azimuth compression, and two-dimensional peak localization.

#### Simulation geometry and signal model

A canonical point target with reflectivity ${\sigma }_{0}$ is placed at true range ${R}_{0}$ and azimuth ${x}_{0}$. The radar platform moves along the azimuth track with constant velocity $v$ while transmitting the waveform under test. The instantaneous slant range is

30 $$R\left({t}_{a}\right)= \sqrt{{R}_{0}^{2}+{({x}_{0}+v{t}_{a})}^{2}}$$

where ${t}_{a}$ is the azimuth (slow-time) variable.

The received echo is modeled as,

31 $${s}_{r}\left(t,{t}_{a}\right)= {\sigma }_{0}{s}_{t}\left(t-\frac{2R\left({t}_{a}\right)}{c}\right)$$

where ${s}_{t}(t)$ is the exact waveform used in transmission. For consistency with the proposed method, the transmitted signal is the optimized piecewise NLFM waveform defined in (4)–(12), with instantaneous frequency,

32 $${f}_{i}\left(t\right)= {f}_{q} \mathrm{for} t\in \left[{t}_{q-1},{t}_{q}\right]$$

And accumulated phase,

33 $${\theta }_{i}\left(t\right)=2\pi \left[{\frac{\mathrm{A}}{3}(t-{T}_{B})}^{3}+ \frac{B-A{T}^{2}}{2T}{(t-{T}_{B})}^{2}+{f}_{B }(t-{T}_{B})\right], {T}_{B}\le t\le {T}_{E}$$

This ensures that the point-target simulation truly reflects the spectral shaping introduced by the proposed PWL-NLFM sweep.

#### Two-dimensional SAR processing

The received echo is processed using range-then-azimuth compression as described in the following subsections.

##### Range (fast-time) compression

The impulse response of the matched filter using the complex conjugate of the same NLFM waveform employed in transmission can be expressed as,

34 $${h}_{r}\left(t\right)={\it{s}}_{t}^{*}\left(-t\right)$$

##### Azimuth (slow-time) compression

A standard Doppler-domain matched filter is applied with the transfer function,

35 $${H}_{a}\left({f}_{a}\right)=\mathrm{exp}\left(-j\pi \frac{{f}_{a}^{2}\lambda {R}_{0}}{{v}^{2}}\right)$$

This produces a fully focused 2-D point-target response.

The resulting image $I(\tau ,x)$ is used to compute PSLR, ISLR, and IRW.

#### Definitions and computation of PSLR, ISLR, and IRW

From the focused 2-D response, the metrics are extracted along the range dimension.

##### Peak sidelobe level ratio (PSLR)

PSLR is the ratio of the amplitude of the highest sidelobe to the mainlobe peak. For 2-D point target image, it can be numerically evaluated as follows,

36 $$\mathrm{PSLR}=10{\mathrm{log}}_{10}\left[\frac{{\mathrm{max}}_{i\ne 0}\left|I\left({\tau }_{i},{x}_{0}\right)\right|}{\left|I\left({\tau }_{0},{x}_{0}\right)\right|}\right]$$

A lower PSLR indicates more effective sidelobe suppression, which reduces false detections in SAR imagery.

##### Integrated sidelobe ratio (ISLR)

ISLR measures the total energy contained in the sidelobes relative to the mainlobe. It can be numerically evaluated as follows.

37 $$\mathrm{ISLR}=10{\mathrm{log}}_{10}\left[\frac{{\sum }_{\tau \in sidelobes}{\left|I\left(\tau ,{x}_{0}\right)\right|}^{2}}{{\sum }_{\tau \in mainlobe}{\left|I\left(\tau ,{x}_{0}\right)\right|}^{2}}\right]$$

Lower ISLR values reflect better energy concentration in the mainlobe, improving image contrast.

##### Impulse response width (IRW)

IRW characterizes the temporal width of the mainlobe, defined as the distance between the first nulls surrounding the peak. Narrower IRW corresponds to higher resolution, while slightly increased IRW may occur as a trade-off for stronger sidelobe suppression.

## Results and discussion

This section presents and discusses some numerical examples to investigate the scheme of signal processing proposed to enhance the detection performance and improve the resolution of the land imaging SAR systems. Yet, first we will present and discuss the performance of the conventional NLFM pulse chirping method based on quadratic time dependence of the instantaneous frequency to be used as a reference for evaluating the performance of the proposed method.

### Conventional second-order NLFM for SAR pulse chirping

**Fig. 8:**
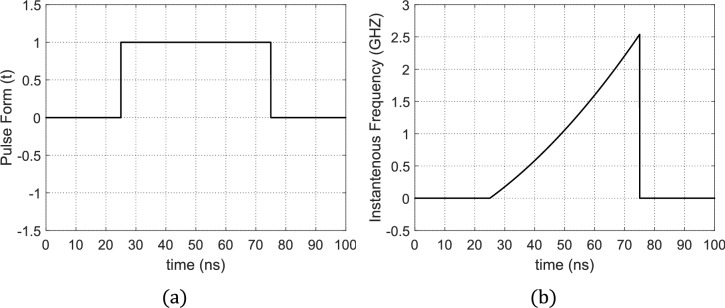
(**a**) The rectangular pulse before being chirped. (**b**) 2nd-order Time–frequency curve as described by expression (6) with $A=3.395\times {10}^{23} {\mathrm{s}}^{-3}$.

**Fig. 9:**
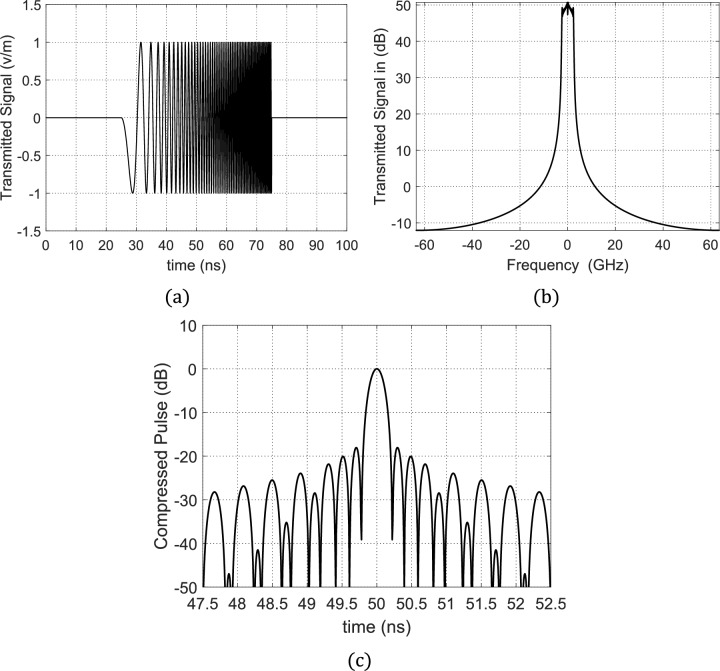
The chirped pulse using 2nd-order curve for NLFM: (**a**) Time-domain form of the chirped pulse, $s(t)$. (**b**) Frequency-domain form of the chirped pulse $S(f)$. (**c**) Time waveform of the compressed pulse at the output of the MF with maximum SLL of about −18.06 dB and PCR of 115.

In the conventional NLFM using 2nd-order frequency-time dependence, the unchirped rectangular pulse that is shown in Fig. 8(a) becomes envelop of a sinusoidal wave. The instantaneous frequency of this wave increases as a 2nd-order curve with time as shown in Fig. 8 (b). If a square pulse of duration ${\rm T}=50 \mathrm{ns}$ is subjected to NLFM using the quadratic time-dependence given by Eq. (6), then the resulting chirped pulse will have the time waveform shown in Fig. 9(a). The application of the results of FFT in the frequency spectrum of the chirped pulse are shown in Fig. 9(b).

The MF process is applied to receive and process the echo pulse, $r(t)$, that is reflected from the radar target because of the incidence of the chirped pulse, $s(t)$, described before. In Fig. 7 the time waveform of the 2nd-order NLFM-chirped pulse, $p(t)$, at the receiver MF output is presented where $p(t)$, has a main lobe and many side lobes and the maximum SLL is about $-18.06\text{ dB}$. The main lobe has very short time duration compared to the duration $T$ of the transmitted radar pulse. The compressed pulse width $p(t)$ can be calculated as the time difference between the first two nulls on both sides of the main lobe. The compressed pulse width is $0.44\text{ ns}$. The PCR can be obtained by dividing the pulse width before compression shown in Fig. 8 (a) by the main lobe width of $p\left(t\right)$ and this gives a PCR of $115$. Hence, the frequency chirping using 2nd-order NLFM (quadratic time-dependence of the instantaneous frequency) leads to very high PCR but the resulting SLL is about $-18 \mathrm{dB}$, which may not be enough for accurate SAR imaging.

### Optimized staircase frequency modulation for pulse compression

**Fig. 10:**
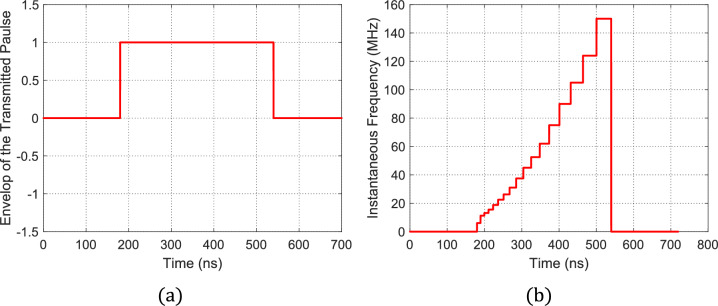
(a) The rectangular pulse before being chirped. (b) 2nd-order Time–frequency curve with $A=3.395\times {10}^{23} {\mathrm{s}}^{-3}$.

**Fig. 11:**
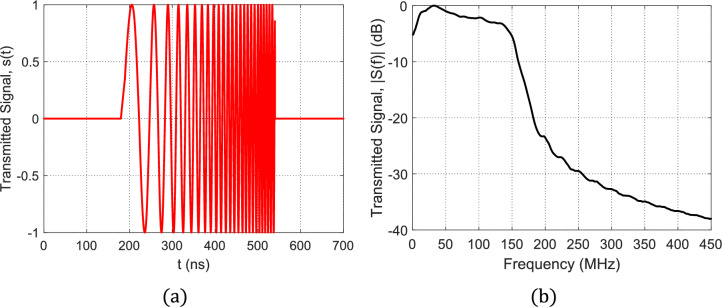
The chirped pulse using 2nd-order curve for NLFM: (**a**) Time-domain form of the chirped pulse, $s(t)$. (**b**) Frequency-domain form of the chirped pulse $S(f)$.

In the Optimized Staircase Frequency Modulation for Pulse Compression, the unchirped rectangular pulse is shown in Fig. 10(a) and the staircase segmentation of the instantaneous frequency as a function of time is shown in Fig. 10 (b), then the resulting chirped pulse will have the time waveform shown in Fig. 11(a). The application of the results of FFT in the frequency spectrum of the chirped pulse is shown in Fig. 11(b).

Then the MF process is applied where the PCR can be obtained by dividing the pulse width before compression shown in Fig. 10 (a) by the main lobe width of $p\left(t\right)$ and this gives a PCR of $50$ and SLL is $-20\text{ dB}$.

### Relation between the achieved CR, SLL and the number of linear segments

The PSO algorithm aims at achieving a predetermined value of the PCR and minimizing the SLL. Due to the computational efficiency of the proposed PSO algorithm, it takes a few iterations to arrive at the optimum value of the SLL and desired PCR. The application of the PSO algorithm results in constructing the staircase segmented curve for the variation of the instantaneous frequency with time, hence decreasing the SLL from its initial value ($-18.06\text{ dB}$) that has been achieved using the conventional 2nd-order frequency chirping method discussed in Sect. “Conventional Second-Order NLFM for SAR pulse chirping”, to much better values.

The number of linear segments used to build the time–frequency curve, $Q$, can be considered as a parameter to get varying values of the PCR and the corresponding SLL. It will be demonstrated from this simulation results that increasing $Q$ results in decreasing the SLL and decreasing the PCR at the same time. Hence, a compromise needs to be made to select the value of $Q$ that achieves the optimization goals of the SAR pulse compression, for example, the maximum PCR can be achieved at $Q=1$. Here, the PSO aims are to minimize the SLL while keeping the PCR as close as possible to 127. This is achieved by the conventional LFM chirping of the rectangular radar pulse. Because the time–frequency curve has only one segment with a slope ${s}_{1}$, there are no control parameters to be utilized by the PSO to get better performance than that obtained by the conventional LFM pulse compression method. The time–frequency curve achieved in this case is shown in Fig. 13(a) and the time waveform of the compressed pulse, $p\left(t\right)$ at the MF output is shown in Fig. 13(b). Here, the obtained SLL is $-13.1\text{ dB}$ and the obtained PCR is 127, that is the well-known performance of the conventional LFM. Note that in this example, the central frequency is 1.27 GHz and the pulse width is $\mathrm{T}=5\text{ ns}$.

**Fig. 12:**
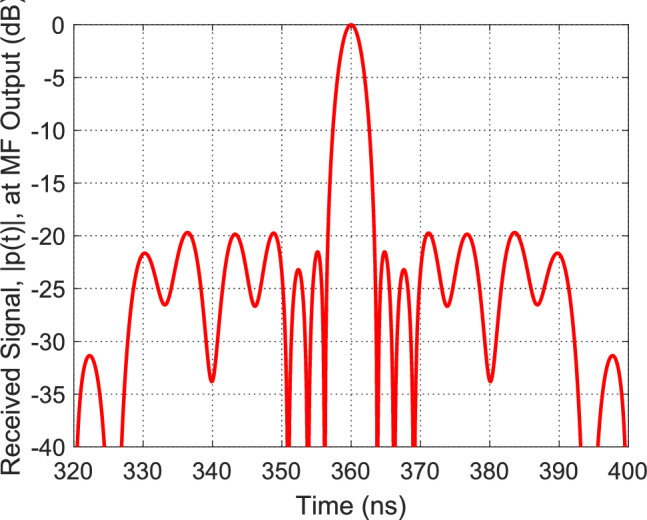
Time waveform of the compressed pulse at the output of the MF with maximum SLL of about −20 dB and PCR of 50.

**Fig. 13:**
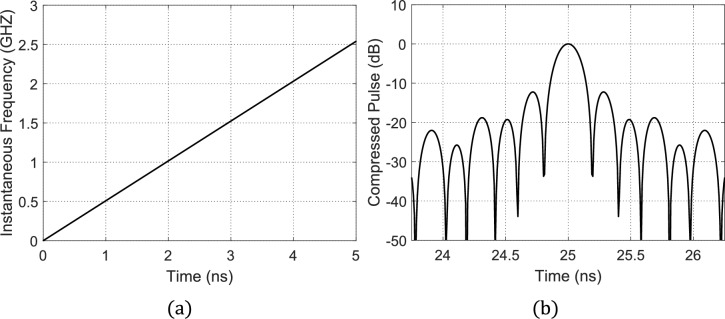
The instantaneous frequency curve of one segment, $Q=1$ (equivalent to LFM) leads to a compressed pulse with maximum SLL of $-13.1\text{ dB}$ and PCR of $127$. (**a**) Optimized instantaneous frequency curve. (**b**) Compressed pulse at the MF output. Note that in this example, the central frequency is 1.27 GHz and the pulse width is $\mathrm{T}=5\text{ ns}$.

**Fig. 14:**
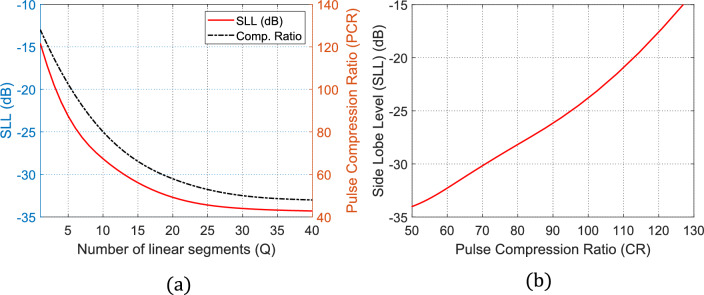
Variation of the SLL and PCR after applying the PSO. (**a**) Dependencies of the SLL and PCR on the number of linear segments of the frequency-time curve. (**b**) Relation between the achievable SLL and the corresponding PCR.

It is shown from the previous examples (Fig. 12 and Fig. 13) that, irrespective of the operating SAR frequency, the PCR decreases with increasing the number of linear segments, $Q$. However, the maximum SLL decreases with increasing $Q$. The relations showing the dependence of the maximum SLL and the corresponding PCR on the number of linear segments, $Q$, are presented in Fig. 14 (a) while Fig. 14 (b) plots the relation between the achievable SLL as a function of the corresponding PCR.

The tunable parameter $Q$ in the proposed PWL-NLFM waveform directly governs the trade-off between pulse compression ratio (PCR) and sidelobe suppression. Higher $Q$ values divide the pulse into more segments, allowing stronger sidelobe suppression but slightly broadening the mainlobe and reducing PCR, which corresponds to a small power reduction in the output pulse. For example, a high PCR of $127$ $(\approx 1.6\%$ power reduction) results in a sidelobe level similar to standard LFM ($\approx -13.1\text{ dB}$), prioritizing ultra-high resolution at the cost of higher sidelobe energy. Conversely, a lower PCR of 50 $(\approx 4\%$ power reduction) achieves a significantly improved SLL of $\approx$−20 dB, yielding a cleaner image with fewer artifacts but slightly lower range resolution.

In practical SAR imaging scenarios, the operational priority depends on the mission requirements. For applications such as tunnel detection, where weak buried targets are often masked by clutter, sidelobe suppression is typically more critical than achieving the absolute highest resolution. A cleaner image reduces false detections and enhances target detectability, which justifies selecting a moderate PCR with lower SLL. For reconnaissance or mapping tasks requiring the finest spatial detail, high PCR and narrow mainlobes may be preferred despite higher sidelobe levels. Therefore, the flexibility provided by the tunable $Q$ parameter allows the SAR operator to adapt the waveform to specific mission needs, balancing resolution and image clarity according to the scene and target characteristics.

### Perfromance comparison for 2-D point-target under different waveforms

To validate the quality of the generated SAR pulses, point-target simulations were performed in both range (fast time) and azimuth (slow time) dimensions. This allows assessment of the transmitted waveforms’ ability to achieve low sidelobes and high resolution under realistic SAR operation. Three representative waveforms were considered:

- Linear FM (LFM), conventional reference chirp.
- Conventional quadratic NLFM, standard second-order non-linear frequency modulation.
- Proposed PWL-optimized NLFM, piecewise-linear, slope-optimized waveform with $Q=20$ segments.

The resulting performance metrics, including Peak-to-Sidelobe Level Ratio (PSLR), Integrated Sidelobe Level Ratio (ISLR), and Impulse Response Width (IRW), are summarized in Table 1. Here, IRW is normalized relative to the mainlobe width of the reference LFM pulse.

**Table 1:** PSLR, ISLR, and IRW comparison for different chirp strategies for Two-dimensional (range–azimuth) point-target response.

Waveform	ISLR (dB)	ISLR (dB)	IRW (normalized)
LFM	–13.2 dB	–8.4 dB	1.00
Classical NLFM (quadratic)	–18.7 dB	–14.5 dB	1.07
Proposed PWL Optimized NLFM($Q=20$)	–33.0 dB	–21.8 dB	1.15

**Fig. 15:**
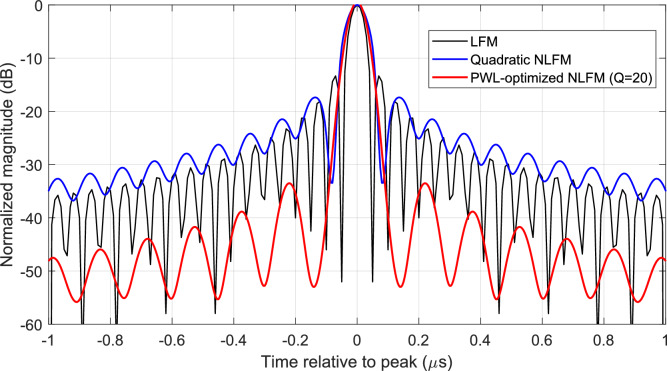
Two-dimensional (range–azimuth) point-target response using the proposed PWL-NLFM waveform. The range profile is extracted along the azimuth of the target and evaluated for PSLR, ISLR, and IRW. The significantly suppressed sidelobes demonstrate the effectiveness of the optimized NLFM sweep.

Figure 15 shows the two-dimensional (range–azimuth) point-target response for the proposed PWL-NLFM waveform. The range profile is extracted along the azimuth of the target, and the corresponding PSLR, ISLR, and IRW values are evaluated. The significantly suppressed sidelobes in both dimensions demonstrate the effectiveness of the optimized NLFM sweep in improving SAR imaging performance compared to conventional linear and quadratic NLFM waveforms.

### Computational efficiency of the proposed PSO algorithm

**Fig. 16:**
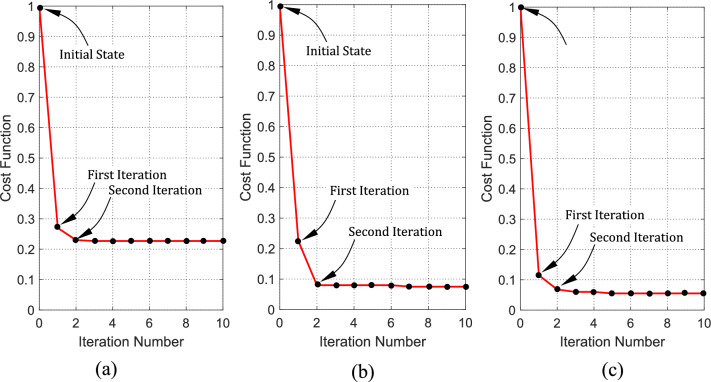
Decay of the cost function with the progressive iterations of the PSO algorithm for the SAR pulse compression cases whose results are presented in Figs. 9, 10, and 11. (**a**) $Q=2$. (**b**) $Q=10$. (**c**) $Q=30$.

The PSO algorithm developed in this work is characterized by its simplicity, computational efficiency and fast convergence. It takes only a few iterations to achieve the optimization goals such that the cost function decays to less than 20% of its initial value (at the beginning of the algorithm) just after the first iteration. A little decay of the cost function can be observed in the second iteration. The decay rate of the cost function for the three cases presented in Sect. “Optimized Staircase Frequency Modulation for Pulse Compression” for $Q=2, 10, 30$ are presented in Fig. 16 a, b, c respectively. This fast decay rate gives the PSO algorithm an advantage over other evolutionary multi-objective optimization techniques.

## Comparative performance with other pulse compression methods

Table 2 summarizes the peak sidelobe level (PSLL) achieved in this work and several recent studies applying different NLFM or pulse-compression techniques. It should be noted that several of the compared works do not provide explicit values for pulse compression ratio (PCR) or power reduction factor (PRF), which limits the possibility of a fully quantitative comparison. Nonetheless, the table allows a qualitative assessment of the relative performance of various approaches.

**Table 2:** Comparisons among the achieved SLL due to the application of SAR pulse compression techniques available in some publications and the SLL achieved in the present work.

Work	Radar Compression Method	Performance Metrics				
		Required Peak Power (% of Uncompressed Pulse)		Pulse Compression Ratio (PCR)		Peak Sidel Lobe Level (PSLL)
^16^	Starring spotlight mode with NLFM	NA		NA		$-22\text{ dB}$
^17^	Distinctive piecewise NLFM modulation sub-carrier	NA		NA		$-27\text{ dB}$
^18^	OFDM based on Piecewise NLFM	NA		NA		$-28\text{ dB}$
^19^	Piecewise NLFM	NA		NA		$-36.6\text{ dB}$
^20^	Optimization using Lagrangian Method	NA		NA		$-38 \mathrm{dB}$
^21^	NLFM based on the ALGA and PWL functions	NA		NA		$-40\text{ dB}$
Proposed	Arbitrarily-defined piece-wise NLFM (Q = 1)	$1.6 \%$		$127$		$-13.1\text{ dB}$
Proposed	Arbitrarily-defined piece-wise NLFM (Q = 10)	$2.5 \%$		$80$		$-28\text{ dB}$
Proposed	Arbitrarily-defined piece-wise NLFM (Q = 40)	$4.0 \%$		$50$		$- 34.7\text{ dB}$

The proposed method achieves competitive sidelobe suppression while offering tunable performance through the number of linear segments $Q$ used in the piece-wise linear time–frequency curve. Specifically, when using $Q=10$ segments, the waveform attains a PSLL of $-28\text{ dB}$ at a PCR of $80$, with the transmitted pulse power after compression reduced to $2.5 \%$ of its original value. Increasing the number of segments to $Q=40$ yields a PSLL of $-34.7\text{ dB}$ at a PCR of $50$, with the transmitted pulse power after compression reduced to $4.0 \%$ of the original pulse.

The peak power required for the compressed pulse is inversely proportional to the pulse compression ratio (PCR). For a given PCR ($\eta$), the required peak power fraction is approximately ${P}_{Peak}/{P}_{Uncompressed}\approx 2/\eta$ (Eq. 28). Hence, for $\eta =127$ the required peak power is only ≈1.6% of the uncompressed pulse, while for $\eta =50$, it is $\approx 4\%$. This representation clarifies the actual power savings achieved by pulse compression and provides a direct measure of the efficiency of the proposed PWL-NLFM waveform.

This means that for $Q=10$ and $40$, the transmitted power is reduced to $4.0\%$ and $2.5\%$, respectively, of its original value before pulse compression, which demonstrates the flexibility of the proposed approach: by adjusting $Q$, users can optimize the trade-off between sidelobe suppression and pulse compression performance.

Some of the existing methods achieve very low PSLL values through combined or advanced optimization techniques, such as the approaches in^19^ and^20^, which report PSLLs of $-36.6\text{ dB}$ and $-38\text{ dB}$, respectively. However, because most of these works do not report PCR or PRF, direct numerical comparison with the proposed method is limited.

The recent study by Wei et al^23^. demonstrates an intermediate-frequency NLFM signal generator for UAV-borne SAR missions. While PSLL and PCR values are not explicitly reported in^23^, its inclusion provides additional context for the development of NLFM generation techniques in airborne SAR. Compared to^23^, the proposed approach targets VHF airborne SAR for buried-object detection, operates under low-power constraints, and enables simultaneous optimization of SLL and PCR, highlighting its practical advantages for power-constrained, high-penetration SAR imaging applications.

## Conclusion

In this paper, a low-power transceiver was proposed for airborne synthetic aperture radar (SAR), targeting high-resolution detection of buried objects, particularly hidden tunnels. The SAR system operates in the VHF band, selected for its capability to penetrate the ground and enable detection of concealed tunnels with sufficient imaging resolution. The proposed receiver incorporates a matched filter (MF) to maximize the signal-to-noise ratio (SNR) at the output without increasing the received signal power.

Frequency chirping is implemented using a staircase piecewise-linear (PWL) time–frequency curve, which is optimized using particle swarm optimization (PSO) to maximize SNR and achieve the desired high resolution without increasing the pulse peak power. A key feature of the proposed method is the tunable parameter $Q$, controlling the number of PWL segments. Adjusting $Q$ allows the waveform to balance sidelobe suppression and pulse compression ratio according to mission requirements: larger $Q$ produces stronger sidelobe reduction for enhanced buried-object detectability (PSLR down to −33.0 dB, ISLR −21.8 dB), whereas smaller $Q$ preserves a narrower mainlobe for higher range resolution.

The received echo has an extended duration and lower instantaneous power compared to an unchirped pulse. The carefully designed impulse response of the receiver ensures that, when convolved with the received signal, a narrow, high-bandwidth pulse is obtained at the output, enhancing imaging resolution while maintaining low-power operation. Two-dimensional point-target simulations confirm the effectiveness of the proposed waveform in suppressing sidelobes and maintaining focus along both range and azimuth, demonstrating its suitability for VHF airborne SAR applications aimed at tunnel detection.

Overall, we have demonstrated a PSO-based method that provides a flexible trade-off between SLL and PCR, allowing system designers to prioritize imaging resolution or sidelobe suppression based on the specific application by adjusting the number of linear segments $Q$.

## Data Availability

The datasets used and/or analysed during the current study available from the corresponding author [Yasser.Siddik, yassersiddik1982@gmail.com] on reasonable request.
